# Extension of the shelf-life of fresh pasta using modified atmosphere packaging and bioprotective cultures

**DOI:** 10.3389/fmicb.2022.1003437

**Published:** 2022-09-02

**Authors:** Marinella Marzano, Maria Calasso, Giusy Rita Caponio, Giuseppe Celano, Bruno Fosso, Domenico De Palma, Mirco Vacca, Elisabetta Notario, Graziano Pesole, Francesca De Leo, Maria De Angelis

**Affiliations:** ^1^Istituto di Biomembrane, Bioenergetica e Biotecnologie Molecolari, Consiglio Nazionale delle Ricerche, Bari, Italy; ^2^Dipartimento di Scienze del Suolo, della Pianta e degli Alimenti, Università degli Studi di Bari “Aldo Moro”, Bari, Italy; ^3^Dipartimento di Bioscienze, Biotecnologie e Biofarmaceutica, Università degli Studi di Bari “Aldo Moro”, Bari, Italy; ^4^Food Safety Lab s.r.l., Corato, BA, Italy

**Keywords:** fresh pasta, modified atmosphere packaging, bioprotective cultures, multi-omics approach, metagenomics, shelf-life

## Abstract

Microbial stability of fresh pasta depends on heat treatment, storage temperature, proper preservatives, and atmosphere packaging. This study aimed at improving the microbial quality, safety, and shelf life of fresh pasta using modified atmosphere composition and packaging with or without the addition of bioprotective cultures (*Lactobacillus acidophilus*, *Lactobacillus casei*, *Bifidobacterium* spp., and *Bacillus coagulans*) into semolina. Three fresh pasta variants were made using (i) the traditional protocol (control), MAP (20:80 CO_2_:N_2_), and barrier packaging, (ii) the experimental MAP (40:60 CO_2_:N_2_) and barrier packaging, and (iii) the experimental MAP, barrier packaging, and bioprotective cultures. Their effects on physicochemical properties (i.e., content on macro elements, water activity, headspace O_2_, CO_2_ concentrations, and mycotoxins), microbiological patterns, protein, and volatile organic compounds (VOC) were investigated at the beginning and the end of the actual or extended shelf-life through traditional and multi-omics approaches. We showed that the gas composition and properties of the packaging material tested in the experimental MAP system, with or without bioprotective cultures, positively affect features of fresh pasta avoiding changes in their main chemical properties, allowing for a storage longer than 120 days under refrigerated conditions. These results support that, although bioprotective cultures were not all able to grow in tested conditions, they can control the spoilage and the associated food-borne microbiota in fresh pasta during storage by their antimicrobials and/or fermentation products synergically. The VOC profiling, based on gas-chromatography mass-spectrometry (GC-MS), highlighted significant differences affected by the different manufacturing and packaging of samples. Therefore, the use of the proposed MAP system and the addition of bioprotective cultures can be considered an industrial helpful strategy to reduce the quality loss during refrigerated storage and to increase the shelf life of fresh pasta for additional 30 days by allowing the economic and environmental benefits spurring innovation in existing production models.

## Introduction

Pasta is a cereal-based food of the traditional Mediterranean diet, popular worldwide due to its convenience, palatability, and nutritional quality, ideal for easy and quick meals (Antognelli, 1980; Arendt and Zannini, 2013; Carini et al., 2014; Zardetto et al., 2021). According to Italian law, “fresh pasta” is defined as the product obtained by extrusion or lamination of a dough made of durum wheat semolina or alternative flours and water, having moisture content between 24% and 30%, water activity (a_w_) between 0.92 and 0.97, and stored at 4 (±2)°C (Dpr, 2001; Costa et al., 2010; Carini et al., 2014). In this form, fresh pasta has on average 2–3 days-long shelf life even though this depends on the microbial cell density found at the end of the production process (Tabanelli, 2020). The heat treated (equivalent to pasteurization) industrial fresh pasta, stored at an appropriate temperature, has a shelf life of 30–90 days (Pagani et al., 2007; Angiolillo et al., 2017). However, its shelf life after packaging depends on microbial survival rates to thermal treatments, overcoming the hurdles determined by thermal treatment, a_w_, and storage temperature (Sanguinetti et al., 2011, 2016). The high moisture content and a_w_, as well as the nutrient content, lead to microbial metabolic activities (Guerzoni et al., 1994; Zardetto, 2005a; Del Nobile et al., 2009a) determining fresh pasta as a foodstuff easily perishable and compromising both safety and sensorial characteristics of the end product (Del Nobile et al., 2009a; Costa et al., 2010; Oliveira et al., 2014). For this reason, based on nationality, different laws allowed for using chemical preservatives and bacteriostatic compounds, such as potassium sorbate or sodium benzoate, to maintain the microbial safety and quality of fresh or filled pasta (Fda, 2006; Li et al., 2011; Shahmohammadi et al., 2016; Reg. UE 1129/2011).

Nowadays, the broad diffusion of fresh pasta and, particularly, the increased consumers’ demand for reducing the use of synthetic preservatives have provided the interest to extend the product shelf life (90–120 days) taking advantage of “clean-label” methods (Del Nobile et al., 2009b; Angiolillo et al., 2017; Schettino et al., 2019). Innovative approaches are those based on modified atmosphere packaging (MAP) and biopreservatives, both aimed to reduce the growth of microorganisms surviving heat thermal treatments while maintaining the traditional organoleptic properties and assuring the hygienic quality of fresh pasta (Del Nobile et al., 2009a).

Modified atmosphere packaging is a well-established technique for preserving fresh pasta quality by taking advantage of gas ratios surrounding the product that is different from the air (Zardetto, 2005b; Del Nobile et al., 2009a). The ratios 70:30 and 60:40 N_2_:CO_2_ are the most commonly used MAP gas mixtures to preserve the microbial quality of fresh pasta (Zardetto et al., 2022). These ratios limit microbial overgrowth and toxin production reducing the physicochemical deterioration of packaged foods (Zardetto, 2005b; Chaix et al., 2015). The un-/success of MAP depends on the structure, thickness, area, and permeability of the used film for packaging (e.g., thermoformed trays or flexible films containing specific barrier properties), gradient concentrations, differences in pressure across the film, and temperature (Gholizadeh et al., 2007). Furthermore, recent advances in MAP technologies were based on the application of environmentally friendly film materials in fresh food preservation (Qu et al., 2022; Zardetto et al., 2022).

Biopreservation is a biotechnological strategy based on the use of bioprotective cultures (BCs) or their antimicrobials and fermentation products, such as bacteriocins and organic acids, aimed at preserving foods and extending the shelf life in terms of spoilage and pathogen control (Ananou et al., 2007; Oliveira et al., 2018). Lactic acid bacteria (LAB) have antagonistic properties, proven antimicrobial properties, and safe history that make them ideal biopreservative candidates (Ghanbari et al., 2013; Cifuentes Bachmann and Leroy, 2015). In previous works, LAB were added during pasta-making to have a final product with a lower glycemic index, low gluten (Calasso et al., 2018), or higher content of B2 vitamin (Capozzi et al., 2011) than controls. Furthermore, LAB strains were recently used to preserve both the microbial and sensory quality of fresh or filled pasta (Angiolillo et al., 2017; Tabanelli, 2020). Also, spore-forming microorganisms (e.g., *Bacillus* spp.) have been used in research studies to produce heat-treated probiotic pasta (Fares et al., 2015; Konuray and Erginkaya, 2020).

Based on these considerations, this work explored two different fields. First, different MAP conditions (gas concentration and packaging materials) have been tested. As a second-step evaluation, the experimental MAP was combined with a multi-strain probiotic mixture acting as BCs during pasta production. Therefore, both were compared against control pasta to assess differences in chemical, microbiological, and metabolomic parameters considering also temporal dynamics (storage) based on a multi-omics approach. Hence, the microbiota was profiled through culturomics and rDNA gene-target metagenetics, while chemical, proteomic, and metabolomic profiles of the fresh pasta samples have been also studied.

## Materials and methods

### Pasta making

Fresh pasta samples were produced in a semi-industrial factory (Altamura, Bari, Italy), in duplicate on three consecutive days, using commercial durum wheat semolina (*Triticum turgidum* L. var. *durum*; moisture 14.6 ± 1.3 g/100 g, ash 0.63 ± 0.06 g/100 g, proteins 13.8 ± 0.6g/100 g, dry gluten 11.2 g/100 g, all on dry weight). All samples were prepared in a semi-industrial plant equipped with a bronze die. The chosen shape of pasta was a short, thin twisted pasta type, named “trofie.”

**FIGURE 1:**
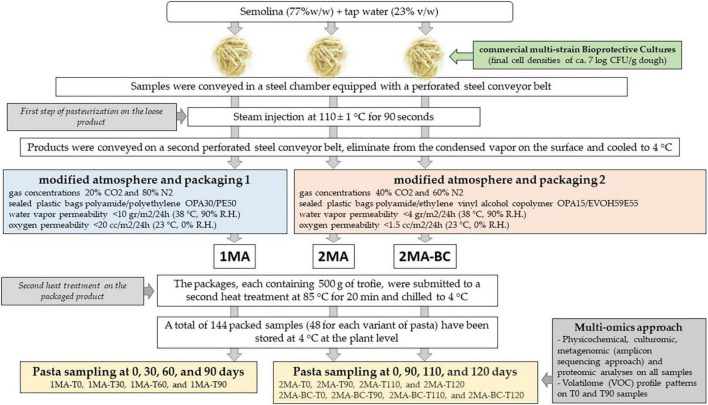
Schematic design showing the production of fresh pasta at the semi-industrial factory and the analyses performed.

Three different sets of pasta were manufactured (Figure 1 and Appendix A): (i) control fresh pasta made following the conventional protocol and MAP (1MA); (ii) fresh pasta obtained by conventional protocol and packaged in experimental MAP (2MA); and (iii) fresh pasta made with the addition of bioprotective cultures (BCs) and packaged in experimental MAP (2MA-BC).

In 2MA-BC, a commercial freeze-dried multi-strain probiotic mixture commercially available was used. The mixture contained *Lactobacillus acidophilus*, *Bifidobacterium animalis*, *Lacticaseibacillus paracasei* (basonym *Lactobacillus paracasei*), *Lacticaseibacillus casei* (basonym *Lactobacillus casei*), and *Bacillus coagulans* (Montefarmaco OTC SpA, Bollate, Milan, Italy). Freeze-dried BCs were added to semolina (final cell density for each strain ∼7 log CFU/g). BCs were prepared by dissolving lyophilized microorganisms in a liquid contained in the provided bottle immediately before using, as recommended by the manufacturer’s instructions, and added to semolina and water during pasta dough-making (final cell densities ∼7 log CFU/g dough).

For 1MA batches, the sampling was performed at 0, 30, 60, and 90 days (actual shelf-life) of storage at 4 ± 2°C. For both 2MA (2MA and 2MA-BC) batches, the sampling was performed at 0, 90, 110, and 120 days (expected shelf-life) of storage at 4 ± 2°C. All samples were shipped to the laboratory under refrigerated conditions (∼4°C) and immediately chemically and microbiologically analyzed. An aliquot of each sample was frozen (-80°C) until metagenomic and metabolomic analyses were performed.

### Chemical characterization

Lipids, proteins (total nitrogen × 6.25) (Reg. CE 1169/2011), ash, and total dietary fibers were assessed according to AOAC methods 945.38F, 979.09, 923.03, and 991.43 (AOAC International, 1990, 2006), respectively. Moisture content was determined by an automatic moisture analyzer at 105°C (Mod. MAC 110/NP, Rodwang Wagi Elektroniczne, Poland). The carbohydrate content was determined by difference [100 − (moisture + proteins + lipids + ash)]. The energy was determined by multiplying the protein and carbohydrate contents by their calorific value (4 kcal/100 g), while the fat amount by its calorific value (9 kcal/100 g). Water activity in fresh pasta samples was determined according to ISO 18787:2017 method.

Headspace changes of O_2_ and CO_2_ concentrations were measured in packaged samples according to L-MI056 rev.0 Ed.2018 internal method. In detail, the LMI056 method was based on measurements with electrochemical cells of the packaging headspace in terms of gas (percentage) concentrations by a DANSENSOR instrument (Ametek MOCON, Inc., Brooklyn Park, MN, USA). Additionally, total aflatoxins (AFTs), deoxynivalenol (DON), ochratoxin A (OTA), and zearalenone (ZEA) were assessed according to the L-MI067 rev.0 2020 method, which was previously accredited by the Italian certification organization Accredia, based on measurements with SCIEX model 5500 + HPLC-MS/MS (AB Sciex LLC, Framingham, MA, USA) using an isotopically labeled internal standard.

### Cultivable microbiota characterization

Microbiological analyses enumerated total aerobic mesophilic bacteria (UNI EN ISO 4833-1:2013 method), mesophilic lactic acid bacteria (LAB) (ISO 15214:1998), *Bifidobacterium* spp., spore-forming bacteria (SFB), presumptive *B. coagulans*, coliform bacteria (ISO 4832:2006 method), beta-glucuronidase-positive *Escherichia coli* (ISO 16649-2:2001 method), *Enterobacteriaceae* (ISO 21528-2:2017 method), *Salmonella* spp. (AFNOR BIO 12/32-10/11 method), coagulase-positive *Staphylococcus* (UNI EN ISO 6888-2:2004 method), *Listeria monocytogenes* (AFNOR BIO 12/27-02/10 method), *Clostridium perfrigens* (ISO 7937 method), yeasts, and molds (standard method ISO 21527-2:2008 method). The sample preparation was performed in accordance with UNI EN ISO 6887-1:2000 and UNI EN ISO 6887-4:2012. Aliquots (10 g) of semolina and fresh pasta were aseptically removed from each package and homogenized [90 ml of Buffered Peptone Water (BPW)] for total counts of aerobic mesophilic bacteria at 30°C, LAB, bifidobacteria, SFB and *Bacillus*, coliforms, β-glucuronidase-positive *E. coli*, coagulase-positive *Staphylococcus*, *L. monocytogenes*, sulfite reducing clostridia, yeasts, and molds. The determination of *Enterobacteriaceae* and *Salmonella* spp. was carried out in 25 g of sample homogenized in 225 mL BPW. For each sample, appropriate dilutions were performed. *Bifidobacterium* was enumerated according to Tharmaraj and Shah (2003), using MRS agar which is added 0.05% L-cysteine hydrochloride, 0.1 g/L neomycin sulfate, 0.15 g/L nalidixic acid, 3 g/L lithium chloride, and 0.2 g/L paromomycin sulfate, and then plates were anaerobically incubated (37°C for 72 h). To determine SFM densities, dilutions were heat-treated (90 °C for 10 min in a water bath) and spread-plated on nutrient agar plates, then incubated (at 37 °C for 48 h). For the enumeration of presumptive *B. coagulans*, dilutions were heat-treated (75 °C for 30 min), spread-plated on glucose yeast extract agar, and incubated (40 °C for 48–72 h) as described by Konuray and Erginkaya (2020). Except for bifidobacteria, SFB, and *Bacillus*, all used methods were validated and accredited by the Italian certification organization Accredia. All used culture media were previously controlled in accordance with the UNI EN ISO 11133:2014. For each of the following parameters, productivity, selectivity, and specificity scores were considered.

### DNA extraction, sequencing, and bioinformatics analysis

Total genomic DNA was extracted from 1MA, 2MA, and 2MA-BC pasta samples at different time points. About 5 g of each pasta sample was homogenized with 45 ml of sterile saline solution (Minervini et al., 2010). Pellet was treated with FastPrep (BIO 101, C lsbad, Canada) and DNA was extracted using the FastDNA Spin Kit for Soil (MP Biomedicals, Illkrich, France), following the manufacturer’s instructions. Qualitative and quantitative analyses of the extracted DNA were performed using agarose gel (1%) electrophoresis and the Quant-iTTM PicoGreen^®^ dsDNA Assay Kit (Invitrogen, Carlsbad, CA, USA), respectively. The V5–V6 hypervariable regions of the 16S rDNA and the ITS1 (Internal Transcribed Spacer) region, within the ITS region of the gene locus for ribosomal RNA, were chosen as targets for prokaryotic and fungal characterization, respectively (Chakravorty et al., 2007; Bokulich and Mills, 2013). Amplicon libraries strategy agreed with Manzari et al. (2015). In the first PCR round, the overhang primer pairs, BV5 (Next For), and AV6 (Next Rev) (5′-TCGTCGGCAGCGTCAGATGTGTATAAGAGACAG[ATTAG ATACCCYGGTAGTCC]-3′/5′-GTCTCGTGGGCTCGGAGAT GTGTATAAGAGACAG[ACGAGCTGACGACARCCATG]-3′) were used for the analysis of the V5–V6 regions (Manzari et al., 2015), while BITS (Next For) and B58S3 (Next Rev) (5′-TCGTCGGCAGCGTCAGATGTGTATAAGAGACAG[ACCT GCGGARGGATCA]-3′/5′-GTCTCGTGGGCTCGGAGATGT GTATAAGAGACAG[GAGATCCRTTGYTRAAAGTT]-3′) were used for the ITS1 region (Bokulich and Mills, 2013). Finally, equimolar ratios of the purified amplicons were pooled and subjected to 2 × 250 bp paired-end sequencing on the Illumina MiSeq platform. To increase the genetic diversity, the phage PhiX genomic DNA library was added to the mix and co-sequenced (Kozich et al., 2013). The V5--V6 hypervariable regions and ITS1 raw sequencing data were initially quality checked by using FastQC^1^ and multiQC (Ewels et al., 2016). Illumina adapters and PCR primers were removed from raw reads by applying cutadapt (Martin, 2011). The 16S rRNA and ITS1 data were analyzed by using two different pipelines: QIIME2 (Bolyen et al., 2019) and BioMaS (Fosso et al., 2015). The first one relies on ASVs (Amplicon Sequence Variants) estimation and classification, and the latter one performs the taxonomic classification of Illumina data, especially for barcodes characterized by a wide variation in length (i.e., ITS) that may not properly be profiled by using ASV-based approaches. Trimmed V5-V6 data were analyzed by using the QIIME2 suite (version 2019.7). Fastq files were imported as QIIME2 artifacts by using the tools plugin and denoised into ASVs (Callahan et al., 2017) by applying DADA2 (Callahan et al., 2016). The obtained ASVs were taxonomically annotated by using the fit-classifier-sklearn (Pedregosa et al., 2011) plugin and the release 132 of the SILVA database (Pruesse et al., 2007) as the 16S rRNA reference collection and taxonomy. Trimmed ITS1 paired-end reads were merged into consensus sequences using PEAR (version 0.9.6) (Zhang et al., 2013) and then dereplicated through VSEARCH (version 2.15) (Rognes et al., 2016). The unmerged reads were trimmed of low-quality regions (Phred score cutoff of 25), and paired ends containing reads shorter than 50 nt were removed. Both the merged and the unmerged sequences were mapped against the ITSoneDB database (release 141.1) (Santamaria et al., 2012, 2018) by using Bowtie 2 (version 2.3.5.1). The mapping data were filtered according to query coverage (≥70%) and similarity percentage (≥97%) and taxonomically classified by applying TANGO (Alonso-Alemany et al., 2014; Fosso et al., 2018) on the NCBI taxonomy (Kim et al., 2013).

### Protein characterization

Protein fractions (albumins and globulins, gliadins, and glutenins) were extracted from pasta following the method originally described by Osborne (1907) and further modified by Weiss et al. (1993) and Rizzello et al. (2007). All extracts were stored at -80°C until they were used. The protein concentration of the various fractions was determined by the Bradford method (Bradford, 1976).

Aliquots of ca. 15 μg of protein from extracted fractions were separated by sodium dodecyl sulfate-polyacrylamide gel electrophoresis (SDS-PAGE) according to the Laemmli protocol (Laemmli, 1970).

Two-dimensional electrophoresis (2-DE) of ca. 30 μg of proteins was carried out with the Immobiline-polyacrylamide system as previously described (Bjellqvist et al., 1993). The second dimension was carried out in a Laemmli system (Laemmli, 1970). Gels were silver stained, and spot intensities were normalized (Bini et al., 1997). Three gels from each sampling time were analyzed.

### Profiles of volatile organic compounds

To profile volatile organic compounds (VOC)by gas chromatography–mass spectrometry (GC-MS) analyses, 7.25 g of crushed fresh pasta was placed into a 20-ml vial with 5 μl of internal standard 4-methyl-2-pentanol (final concentration of 0.67 μg/g) (Cavallo et al., 2017). A PAL COMBI-xt autosampler (CTC combiPAL, CTC Analysis AG, Zwingen, Switzerland) was used to standardize the extraction procedure. To optimize the headspace solid phase microextraction (HS-SPME), a divinylbenzene/carboxen/polydimethylsiloxane (DVB/CARB/PDMS) (Supelco, Bellefonte, PA, USA) fiber was exposed to the sample headspace for 60 min at 75°C (Giannetti et al., 2021). Adsorbed molecules were desorbed in the injector with the analytical conditions reported by Montemurro et al. (2020), using a Clarus 680 (PerkinElmer, Beaconsfield, UK) gas chromatography equipped with an Rtx-WAX column (30 m × 0.25 mm i. d., 0.25 μm film thickness) (Restek Superchrom, Milano, Italy) and a single-quadrupole mass spectrometer Clarus SQ8MS (PerkinElmer) detector. Each chromatogram was analyzed for peak identification by comparing (i) the retention time (RT) of the detected compound with those of pure standard for HPLC (Sigma-Aldrich, St. Louis, MO, USA) and (ii) experimental mass spectra with those of the National Institute of Standards and Technology database (NIST/EPA/NIH Mass Spectral Library with Search Program, data version NIST 05, software version 2.0 d). Quantitative data for the identified compounds were obtained by the interpolation of the relative areas versus the internal standard area, expressed as μg/g of 2-methyl-4-pentanol.

### Statistical analyses

Two-way ANOVA was applied on the means of data obtained from three biological replicates (analyzed in duplicate), using the statistical software Statistica 12.5 (TIBCO Software Inc., Palo Alto, CA, USA) for Windows. GraphPad Prism version 8.0.1 (GraphPad Software, San Diego, CA, USA) and Permut-MatrixEN software have been used to display bacterial and fungi abundances. Principal Components Analysis was performed through Xlstat 2014 (Addinsoft, New York, NY, USA).

## Results

### Chemical characterization

1MA pasta was produced using *T. durum* flour and tap water by conventional protocol, packaged in CO_2_:N_2_ = 20:80 PACT30 PE50 MAP system, and used as control. 2MA and 2MA-BC pasta samples were packaged in CO_2_:N_2_ = 40:60 PA15/PEEVOH5/PE60 MAP system. 2MA-BC pasta was produced using the commercial spray-dried probiotic bioprotective cultures (BCs) added to pasta dough.

Pasta packaged in experimental MAP with or without the addition of BCs were not different in terms of energy value, proteins, carbohydrates, fibers, fats, and ashes content than 1MA samples (Table 1).

**TABLE 1:** Chemical composition during storage at 4°C of fresh pasta “trofie.”

Parameters	1MA-T0	1MA-T30	1MA-T60	1MA-T90	2MA-T0	2MA-T90	2MA-T110	2MA-T120	2MA-BC-T0	2MA-BC-T90	2MA-BC-T110	2MA-BC-T120
Energy value (kcal/100 g)	354 ± 7.1	354 ± 7.1	354 ± 7.1	354 ± 7.1	355 ± 7.1	355 ± 7.1	355 ± 7.1	355 ± 7.1	355 ± 7.1	355 ± 7.1	355 ± 7.1	355 ± 7.1
Proteins (g/100 g)	10.8 ± 0.22	10.9 ± 0.22	10.9 ± 0.22	10.9 ± 0.22	10.1 ± 0.20	10.3 ± 0.26	10.3 ± 0.23	10.3 ± 0.18	10.6 ± 0.23	10.3 ± 0.19	10.3 ± 0.21	10.2 ± 0.2
Carbohydrates (g/100 g)	52.12 ± 1.04	52.92 ± 1.06	52.1 ± 1.04	51.8 ± 1.04	53.65 ± 1.07	53.85 ± 1.08	55.54 ± 1.11	53.44 ± 1.07	53.92 ± 1.08	53.22 ± 1.06	54.12 ± 1.08	50.52 ± 1.0
of which sugars (g/100 g)	0.30 ± 0.01	0.33 ± 0.01	0.37 ± 0.01	0.32 ± 0.01	0.37 ± 0.01	0.37 ± 0.01	0.40 ± 0.01	0.35 ± 0.01	0.35 ± 0.01	0.34 ± 0.01	0.36 ± 0.01	0.30 ± 0.01
Fibers (g/100 g)	3.0 ± 0.06	3.0 ± 0.04	3.0 ± 0.01	3.0 ± 0.0	3.0 ± 0.01	3.0 ± 0.02	3.0 ± 0.0	3.0 ± 0.06	3.0 ± 0.06	3.0 ± 0.05	3.0 ± 0.05	3.0 ± 0.05
Fats (g/100 g)	1.5 ± 0.03	1.5 ± 0.03	1.5 ± 0.03	1.5 ± 0.03	1.46 ± 0.03	1.46 ± 0.03	1.46 ± 0.03	1.46 ± 0.03	1.48 ± 0.03	1.48 ± 0.03	1.48 ± 0.03	1.48 ± 0.0
of which saturated (g/100 g)	0.5 ± 0.01	0.5 ± 0.01	0.5 ± 0.01	0.5 ± 0.01	0.5 ± 0.01	0.5 ± 0.01	0.5 ± 0.01	0.5 ± 0.01	0.5 ± 0.01	0.5 ± 0.01	0.5 ± 0.01	0.5 ± 0.01
Ashes (g/100 g)	0.98 ± 0.02	0.98 ± 0.02	1.01 ± 0.02	1.00 ± 0.02	0.99 ± 0.02	0.99 ± 0.02	1.00 ± 0.02	1.00 ± 0.02	1.01 ± 0.02	1.00 ± 0.02	1.01 ± 0.02	1.01 ± 0.0
Moisture (g/100 g)	31.6 ± 0.63	30.7 ± 0.61	31.5 ± 0.63	31.8 ± 0.64	30.8 ± 0.62	30.4 ± 0.61	28.7 ± 0.57	30.8 ± 0.62	30 ± 0.60	31 ± 0.62	30.1 ± 0.60	33.8 ± 0.7
A_w_	0.92 ± 0.02^b^	0.95 ± 0.02^a^	0.94 ± 0.02^a^	0.95 ± 0.02^a^	0.93 ± 0.02^a^	0.92 ± 0.01^a^	0.92 ± 0.01^a^	0.92 ± 0.02^a^	0.92 ± 0.01^a^	0.92 ± 0.02^a^	0.92 ± 0.02^a^	0.92 ± 0.02^a^
pH	6.00 ± 0.12^a^	6.02 ± 0.12 ^a^	6.02 ± 0.09 ^a^	6.02 ± 0.11 ^a^	5.99 ± 0.02 ^a^	5.95 ± 0.12 ^a^	5.96 ± 0.12 ^a^	5.96 ± 0.12 ^a^	5.92 ± 0.12	5.9 ± 0.12	5.9 ± 0.12	5.91 ± 0.12
O_2_ (%)	2.3 ± 0.05^c^	2.8 ± 0.06^b^	1.3 ± 0.03^d^	20.1 ± 0.40^a^	0.2 ± 0.00^e^	0.4 ± 0.01^e^	0.3 ± 0.01^e^	0.2 ± 0.00^e^	0.3 ± 0.01^e^	0.3 ± 0.01^e^	0.4 ± 0.01^e^	0.8 ± 0.02^e^
CO_2_ (%)	21.1 ± 0.42^b^	17.4 ± 0.35^c^	17.3 ± 0.35^c^	2.2 ± 0.04^d^	32.6 ± 0.65^a^	38 ± 0.76^a^	39.1 ± 0.78^a^	39.8 ± 0.80^a^	35.6 ± 0.71^a^	38.5 ± 0.77^a^	38.3 ± 0.77^a^	37.2 ± 0.74^a^
Afs (μg/Kg t.q.)^§^	0.1	0.1	0.1	0.1	0.1	0.1	0.1	0.1	0.1	0.1	0.1	0.1
DON (μg/Kg t.q.)^§^	50	67	53	73	55	64.5	54	51.5	61	54	53.5	50
OTA (μg/Kg t.q.)^§^	0.5	0.5	0.5	0.5	0.5	0.5	0.5	0.5	0.5	0.5	0.5	0.5
ZEN (μg/Kg t.q.)^§^	10	10	10	10	4	4	4	10	4	4	4	10

AFs, total aflatoxins; DON, deoxynivalenol, OTA, ochratoxin A; ZEN, zearalenone; Cd, cadmium; Pb, lead. The data are the means of three independent experiments ± standard deviations (*n* = 3). ^§^ The SD of these compounds was in accordance with the official reports from Accredia, which indicated the standard reproducibility and repeatability scores. Means within rows with different letters are significantly different (*p* < 0.05, one-way ANOVA and Tukey’s HSD test).

1MA, control fresh pasta obtained by protocol and packaging MAP conditions used at plant level analyzed at the beginning, after 30 and 60 days, and at the end of the actual 90 days of shelf life (1MA-T0, 1MA-T30, 1MA-T60, 1MA-T90); 2MA, fresh pasta obtained by conventional protocol and packaged in experimental MAP conditions analyzed at the beginning and the end of the actual and expected 90, 110, and 120 days of shelf life (2MA-T0, 2MA-T90, 2MA-T110, 2MA-T120); 2MA-BC, fresh pasta obtained by the addition of bioprotective culture onto semolina and packaged in experimental MAP conditions, analyzed at the beginning and the end of the actual and expected 90, 110, or 120 days of shelf life (2MA-BC-T0, 2MA-BC-T90, 2MA-BC-T110, 2MA-BC-T120).

Compared to the control 1MA, 2MA, and 2MA-BC pasta showed similar levels of moisture content at T0 (31.6, 30, and 30.8%, respectively).

The a_w_ was similar (ca. 0.92) between 2MA and 2MA-BC samples and no differences were found during storage. In contrast, in 1MA, the a_w_ was lowest at the beginning of storage (0.92) while it increased (*p* < 0.05) up to 0.95 at 90 days.

At T0, 1MA, 2MA, and 2MA-BC had pH values of 6.00 ± 0.14, 5.99 ± 0.02, and 5.92 ± 0.12, respectively. No statistical differences (*p* > 0.05) were found during storage.

The headspace gas analysis in 1MA samples showed some changes during storage. In particular, the O_2_ concentration increased from 2.3% (at T0) to 20.1% (at T90) while, concerning the same mentioned time-points of storage, the CO_2_ decreased from 21.1% to 2.2%. Compared to 1MA samples, both 2MA (with or without the addition of probiotic BCs) showed the same percentage (*p* < 0.05) of gas concentration during 120 days of storage, and non-significant differences (*p* > 0.05) were observed between samples.

In all pasta samples, values of mycotoxins (AFTs, DON, OTA, and ZEN) were below the safety limits according to the Regulation (CE) N. 401/2006, and non-significant differences (*p* < 0.05) were found between sample types as well as based on storage.

### Cultivable microbiota of fresh pasta

The baseline cell densities of semolina flour used for pasta manufacturing were higher than final products, mainly due to total aerobic mesophilic microorganisms at 30°C (ca. 3.80 log CFU/g), coliforms (ca. 2.5 log CFU/g), and *Enterobacteriaceae* (ca. 2.76 log CFU/g). Moreover, in the used semolina flour LAB were 1.6 log CFU/g, whereas *Salmonella* and *L. mononocytogenes* were absent in 25 g of samples. Beta-glucuronidase-positive *E. coli*, sulfite-reducing clostridia, and coagulase-positive *Staphylococcus* densities were below the detection limit (<1 log CFU/g). Yeasts and molds were <1 and 2.08 log CFU/g, respectively.

In the experimental trial, the use of experimental MAP conditions with or without the addition of BCs (2MA-BC and 2MA, respectively) was carried out and compared against the 1MA product at the beginning and during 90 or 120 days of storage at 4°C.

Pathogens (*Salmonella* and *L. monocytogenes*) were absent in all samples. Beta-glucuronidase-positive *E. coli*, sulfite-reducing clostridia, and coagulase-positive *Staphylococcus* densities were below the detection limit (<1 log CFU/g) in all samples. Coliforms and *Enterobacteriaceae* densities were below 1 log CFU/g in all samples during the monitored 120 days.

In 1MA, total aerobic mesophilic microorganisms at 30°C grew from 3.00 to 4.34 log CFU/g after 90 days of storage (Table 2). When 2MA condition without BCs was used, aerobic mesophilic cell density was ca. 2.52 log CFU/g and remain almost constant until 120 days of storage. In 2MA-BCs, total aerobic microorganisms were <2.0 log CFU/g during storage.

**TABLE 2:** Viable cell counts (log CFU/g) of different microbial groups during storage at 4°C of fresh pasta “trofie.”.

Microbial group	1MA-T0	1MA-T30	1MA-T60	1MA-T90	2MA-T0	2MA-T90	2MA-T110	2MA-T120	2MA-BC-T0	2MA-BC-T90	2MA-BC-T110	2MA-BC-T120
Aerobic mesophilic microorganisms at 30°C	3.00 ± 0.2^b^	1.64 ± 0.2^d^	2.01 ± 0.2^c^	4.34 ± 0.4^a^	2.52 ± 0.2^b^	2.52 ± 0.2^b^	2.56 ± 0.3^b^	2.64 ± 0.3^b^	<2.00^c^*	<2.00^c^	<2.00^c^	<2.00^c^
Mesophilic lactic acid bacteria	1.95 ± 0.1^b^	<1.00^c^**	<1.00^c^	3.47 ± 0.4^a^	<1.00^c^	<1.00^c^	<1.00^c^	<1.00^c^	<1.00^c^	<1.00^c^	<1.00^c^	<1.00^c^
Spore-forming bacteria	1.5 ± 0.1^b^	1.35 ± 0.3^b^	1.35 ± 0.2^b^	1.32 ± 0.1^b^	1.7 ± 0.12^b^	1.75 ± 0.1^b^	1.55 ± 0.2^b^	1.50 ± 0.2^b^	5.18 ± 0.1^a^	5.31 ± 0.1^a^	5.23 ± 0.2^a^	5.51 ± 0.11^a^
Yeasts	<1.00^b^	<1.00^b^	<1.00^b^	3.09 ± 0.1^a^	<1.00^b^	<1.00^b^	<1.00^b^	<1.00^b^	<1.00^b^	<1.00^b^	<1.00^b^	<1.00^b^
Molds	<1.00^b^	1.12 ± 0.2^ab^	1.40 ± 0.2^a^	1.60 ± 0.2^a^	<1.00^b^	<1.00^b^	<1.00^b^	<1.00^b^	<1.00^b^	<1.00^b^	<1.00^b^	<1.00^b^

Shown are mean values ± standard deviations for fresh pasta samples, analyzed in triplicate.

*Below 2 log CFU/g. To apply ANOVA, these values have been numerically treated as 2.

**Below detection limit (1 log CFU/g). To apply ANOVA, these values have been numerically treated as 1. Means within rows with different letters are significantly different (*p* < 0.05, one-way ANOVA and Tukey’s HSD test).

1MA, control fresh pasta obtained by protocol and packaging MAP conditions used at plant level analyzed at the beginning, after 30 and 60 days, and at the end of the actual 90 days of shelf life (1MA-T0, 1MA-T30, 1MA-T60, 1MA-T90); 2MA, fresh pasta obtained by conventional protocol and packaged in experimental MAP conditions analyzed at the beginning and the end of the actual and expected 90, 110, and 120 days of shelf life (2MA-T0, 2MA-T90, 2MA-T110, 2MA-T120); 2MA-BC, fresh pasta obtained by the addition of bioprotective culture onto semolina and packaged in experimental MAP conditions, analyzed at the beginning and the end of the actual and expected 90, 110, or 120 days of shelf life (2MA-BC-T0, 2MA-BC-T90, 2MA-BC-T110, 2MA-BC-T120).

The LAB cell densities in 1MA at the beginning of refrigerated storage was ca. 1.95 ± 0.1 log CFU/g. In 1MA, LAB increased during 90 days of storage and reached 3.47 log CFU/g, while for samples stored with the innovative gas concentration and film (with or without the addition of BCs), it was <1 log CFU/g until the end of 120 days of storage, without significant differences (*p* < 0.05) between samples (Table 2). Bifidobacteria were not detected in all samples.

The range of counts for SFB and *Bacillus* spp. were similar in all samples analyzed (Table 2). 1MA and 2MA showed low counts of aerobic spore-forming bacteria (1.5 and 1.7 log CFU/g, respectively), without significant differences (*p* > 0.05) during storage, whereas 2MA-BC showed higher (*p* < 0.05) counts of spore-forming bacteria than both 1MA and 2MA.

Yeast and mold densities were below the detection limit (1 log CFU/g) in 2MA and 2MA-BC without significant differences (*p* > 0.05) driven by the addition of BCs. In the 1MA, yeasts and molds grew up to 3.09 and 1.6 log CFU/g, respectively, at 90 days of storage (Table 2 and Supplementary Figure 1). 2MA and 2MA-BC samples did not report either signs of spoilage or microbial alterations until 120 days of storage.

### Proteomic characterization of fresh pasta

No differences (*p* < 0.05) were observed for the concentration of protein fractions in pasta samples, whose levels were stable during storage (data not shown). 1MA pasta protein fractions were separated and visualized in triplicate by SDS-PAGE (Supplementary Figure 2). The intensities of the albumins, globulins, and gliadins bands were similar between the sampling times (Supplementary Figure 2). Protein fractions were also separated and visualized by 2DE. The reproducibility of the 2DE gel performance was analyzed by comparing samples in triplicate (data not shown). Based on SDS-PAGE, no differences were found for the total number of spots and protein expression between samples of 1MA profiled at different times of sampling, indicating the absence of substantial differences or protein degradation on wheat protein fractions of pasta affected by storage. Therefore, no additional samples were investigated in terms of proteomic profiling.

### Study of microbial community dynamics through amplicon sequencing and metagenomics

Profiling the prokaryotic microbiota, nine libraries of dual indexed amplicons of 420 bp related to the V5–V6 hypervariable regions of the 16S rRNA gene were successfully sequenced on the MiSeq platform, using a 2 × 250 bp paired-end (PE) sequencing strategy. No amplicon has been obtained and sequenced for 2MA-BC-T120 pasta samples. All sequenced samples generated reads of high quality with the expected length of 250 bp. Raw sequencing data are available in the SRA repository under the BioProject PRJNA782807. About 88.8% of PE reads were retained as ASVs (Amplicon Sequence Variants), following the denoising procedure, and were subjected to the taxonomic analysis.

**FIGURE 2:**
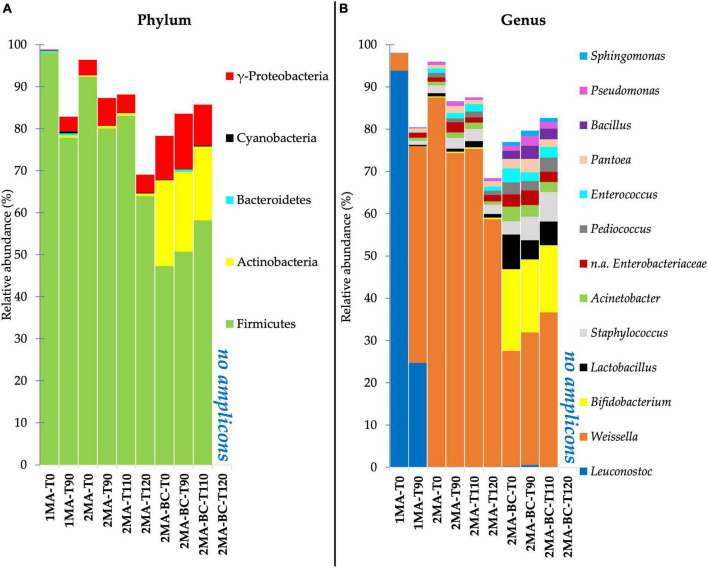
Bacterial community composition at phylum **(A)** and genus **(B)** level for fresh pasta samples by 16S rRNA gene V5–V6 region sequencing. 1MA, control fresh pasta obtained by protocol and packaging MAP conditions used at plant level analyzed at the beginning and the end of the actual 90 days of shelf life (1MA-T0, 1MA-T90); 2MA, fresh pasta obtained by conventional protocol and packaged in experimental MAP conditions analyzed at the beginning and the end of the actual and expected 90, 110, and 120 days of shelf life (2MA-T0, 2MA-T90, 2MA-T110, 2MA-T120); 2MA-BC, fresh pasta obtained by the addition of bioprotective culture onto semolina and packaged in experimental MAP conditions, analyzed at the beginning and the end of the actual and expected 90, 110, or 120 days of shelf life (2MA-BC-T0, 2MA-BC-T90, 2MA-BC-T110, 2MA-BC-T120).

The identified taxa at phylum and genus levels showing a relative abundance higher than 0.1% were reported as stacked bar plots for each analyzed sample (Figure 2). At the phylum level, Firmicutes were dominant in all the samples during storage. Compared to 1MA, 2MA samples with or without the addition of BCs showed a slight increase of gamma-Proteobacteria during storage. Among phyla, 2MA-BC exhibited the presence of Actinobacteria for a relevant proportion of the total microbiota (average abundance of 18% of ASVs from the beginning until the end of the storage). Taxa of the Bacteroidetes and Cyanobacteria were detected in all the samples even though the total relative abundance was lower than 1%.

*Leuconostoc* and *Weissella* were the dominant genera (93.87 and 3.96% of ASVs, respectively) in 1MA-T0 (Figure 2B). *Leuconostoc* was the most relevant genus in 1MA until 90 days of shelf life despite its relative abundance decreasing to 24.7% in 1MA-T90. Instead, *Weissella* increased up to 51.25% in 1MA-T90. *Staphylococcus* and *Enterobacteriaceae* (not assigned at genus level) were ca. 1% till 90 days of storage.

The use of the experimental gas concentration and barrier film combined with the addition of BCs changed the quantitative composition of the prokaryotic microbiota at the genus level. *Weissella* was the most relevant genus despite its relative abundance being ca. 87.5 and 27% of ASVs in 2MA-T0 and 2MA-BC-T0, respectively. The relative abundance of *Weissella* significantly decreased in 2MA-T120 (58.7% of the total ASVs) while increasing up to 37% in 2MA-BC-T110.

In 2MA-BC, the ASVs detection of *Bifidobacterium*, *Lactobacillus*, and *Bacillus* confirmed the presence of the BCs, and these on average were 17.5, 6.12, and 2.55% of the ASVs, respectively, without significant differences determined by storage.

During 120 days of shelf life of samples packaged in 2MA, *Staphylococcus*, *Acinetobacter*, *Pediococcus*, *Enterococcus*, *Pseudomonas*, and *Enterobacteriaceae* (not assigned at genus level) were found each at a lower abundance than 2.85% without significant differences determined by storage.

**FIGURE 3:**
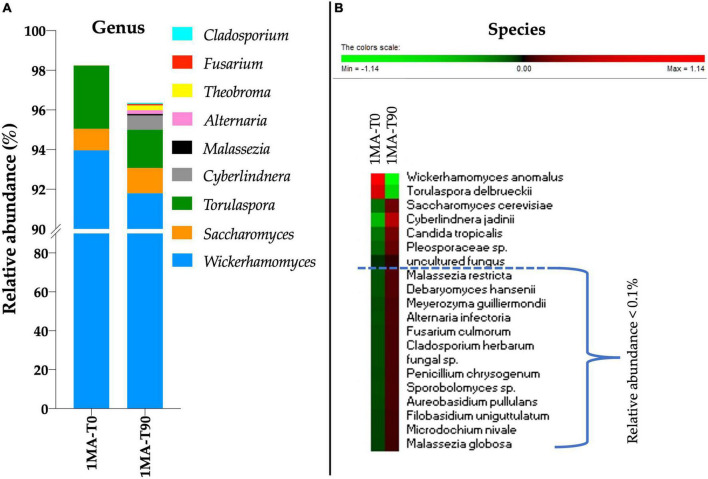
Fungi microbiota (yeasts and molds) composition in fresh pasta samples by ITS1 region sequencing. 1MA, control fresh pasta obtained by protocol and packaging MAP conditions used at plant level analyzed at the beginning and the end of the actual 90 days of shelf life (1MA-T0, 1MA-T90). **(A)** Fungal genera with a relative abundance >0.1% in at least one sample. To note, the scale values started from 90% due to the relative abundance of *Wickerhamomyces* that was >91%. **(B)** Heatmap based on relative abundance (normalized for row) of the top 20 abundant fungal species. Colors correspond to normalized mean data levels from low (green) to high (red).

To profile the fungal microbiota, ITS1 sequencing was performed. No amplicons were retrieved from the total DNA of 2MA and 2MA-BC. Therefore, the ITS1 sequencing was performed only for 1MA samples at the beginning and 90 days of shelf life. *Wickerhamomyces* was the genus more present (94 and 91.7% of the ASVs in 1MA-T0 and 1MA-T90 samples, respectively) (Figure 3A). Besides *Wickerhamomyces*, *Torulaspora* was most represented (3.18 and 1.93% of ASVs in 1MA-T0 and 1MA-T90, respectively) followed by *Saccharomyces* (1.08 and 1.27% of ASVs in 1MA-T0 and 1MA-T90, respectively). *Cyberlindnera*, *Malassezia*, *Alternaria*, *Theobroma*, *Fusarium*, and *Cladosporium* genera had a relative abundance lower than 1% even though a slight increase in their relative abundance was found at T90. The heatmap with normalized values of fungal species’ relative abundance (Figure 3B), including those poor-representative species (0.01–0.1%), showed a more heterogeneous fungal microbiota at T90 than T0.

### Profiling the volatile organic compounds of fresh pasta

**FIGURE 4:**
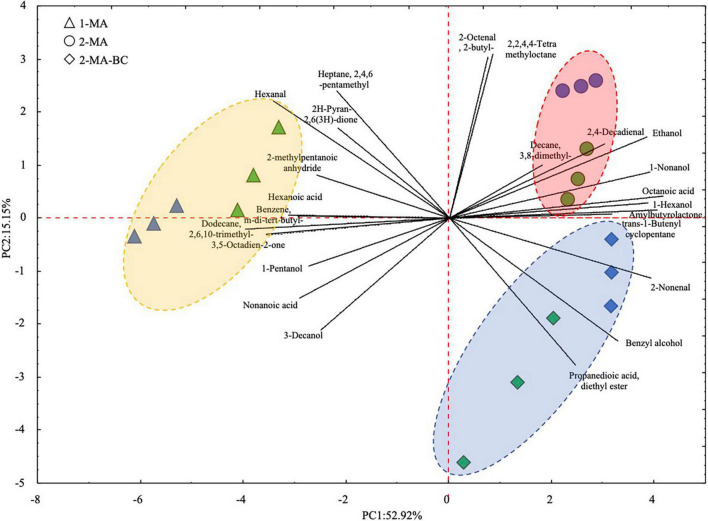
Principal component analysis (PCA) of 24 volatile organic compounds that significantly differed (*p* < 0.05; one-way ANOVA) between fresh pasta samples. ▲, 1MA, control fresh pasta obtained by conventional protocol and packaging MAP conditions used at the plant level; □, 2MA, fresh pasta obtained by conventional protocol and packaged in experimental MAP conditions; ⋄, 2MA-BC, fresh pasta obtained by the addition of bioprotective culture onto semolina and packaged in experimental MAP conditions. Three biological replicates were analyzed at the beginning (0 days, green color) and the end (blue color) of the actual 90 days of storage.

Volatile organic compounds of fresh pasta samples at the beginning and the end of 90 days of refrigerated storage have been investigated by HS-SPME/GC-MS. Forty-two VOCs were detected (Supplementary Table 1). Overall, after 90 days of storage, the total amount of fatty acids and aldehydes in 1MA-T90 was higher compared to 2MA and 2MA-BC pasta samples. The highest concentration of hexanal (*p* < 0.05) was found in 1MA-T90. The highest concentration of total alcohol was observed in 2MA-BC samples. Comparing the different fresh pasta samples, 24 out of 42 VOCs were statistically different. 1MA-T90 showed the highest (*p* < 0.05) amount of hexanal, 2-methylpentanoic anhydride, nonanoic acid, hexanoic acid, 2H-pyran-2,6(3H)-dione, and other hydrocarbons. Both 2MA samples reported a higher concentration (*p* < 0.05) of hexanol and octanoic acid compared to 1MA. Aromatic compounds, benzyl alcohol, and propanedioic acid-diethyl ester were found only in 2MA-BC (Supplementary Table 1). VOC profiles were plotted based on a principal component analysis (PCA) (Figure 4). The PCA plot of samples (score plot) and variables (loading plot) accounted for 68% of the total variance (PC1: 52.9% and PC2: 15.1%). According to PC1, a different VOC profile was observed between 1MA and both 2MA fresh pasta samples. Differently, the PC2 discriminates samples based on both the time of storage and the use of 2MA combined with BCs.

## Discussion

Homemade, artisanal, or industrial fresh pasta products, for example, filled pasta or one heat-treated, are vulnerable under a microbiological point of view based on values of moisture and a_w_. When values of these parameters were too high, they favor the growth of pathogenic or potentially pathogenic microorganisms limiting, therefore, both the healthiness and shelf life of products (Ricci et al., 2017). To preserve fresh pasta and extend its shelf-life avoiding food loss and waste, double-time pasteurization, packaging, temperature of refrigeration, and the use of preservatives are the most studied approaches (Del Nobile et al., 2009a; Costa et al., 2010; Zardetto et al., 2021). With this purpose, the present study describes a “clean-label” approach that uses MAP combined with and without bioprotective cultures (BCs) to increase the shelf life of fresh pasta from 90 to 120 days of storage at 4°C. During storage, several chemical, microbiological, and metabolomic characteristics can be affected by the different technologies that have, in turn, an effect on pasta properties to extend/limit its shelf-life (Costa et al., 2010; Sanguinetti et al., 2011; Tabanelli, 2020; Zardetto et al., 2021). We discovered the suitability of a multi-omics approach to microbial community (bacterial and fungal) profiling through culturomics and metagenomics (amplicon sequencing-based), proteomics, and metabolomics techniques.

First, the influence of the innovative sealed bags and MAP conditions, used in this research, lead to a different O_2_ and CO_2_ concentration during storage being a strong deliverable of the present work, since previous evidence allowed for extended storage thanks to increased concentrations of CO_2_ (Zardetto et al., 2022). Visible molds were found in control fresh pasta packaged under routinary CO_2_:N_2_ (20:80) ratios in PACT30-PE50-bags, in which a decreasing CO_2_ was found after 90 days of storage. Hence, we hypothesized that a gas diffusion between the packaging headspace and the environment was affected by sealed bag characteristics (e.g., mechanical resistance), as suggested by previous works (Lucera et al., 2014; Chaix et al., 2015; Zardetto et al., 2022). In turn, the altered gas composition harbored mold growth on the pasta surface before the use-by date. On the contrary, the experimental CO_2_:N_2_ (40:60) concentration combined with PA15/PEEVOH5/PE60 films for packaging was more stable during storage. Values of WVTR and OTR (Appendix A) of the two films were considered and, as reported in technical sheets, results from permeation tests showed substantial differences. The OTR values particularly differed in terms of magnitude order; the 1MA film being more permeable to O_2_ than bags used for 2MA.

Culture-dependent and -independent approaches allowed for obtaining an integrated overview in terms of microbial dynamics during storage. Microbial cell densities increased during the storage of 1MA samples, which are products no longer marketable after 90 days. A microbial inhibition was found in 2MA based on MAP with 40% CO_2_ in line with previous results (Zardetto, 2005a; Kimbuathong et al., 2020). The CO_2_ permits prolonging of both the lag phase and the time for spoilage inhibiting pivotal enzymatic activities in Krebs’s cycle of microorganisms (Daniels et al., 1985). Instead, in control samples, culturomics seems to indicate a survival rate of several microbial groups despite the heat treatment. Hence, the effectiveness of pasteurization can not be absolute due to the intrinsic and specific characteristics of foods and, as recognized, the shelf life is affected by both the number of surviving microorganisms and textural/structural changes deriving from thermal treatments (Lopez et al., 1998; Tabanelli, 2020). Nonetheless, all used pasta variants were suitable under the hygienic profile, as emphasized by the absence of pathogenic microorganisms (e.g., *Salmonella* spp., *L. monocytogenes*). These outcomes, indeed, are related to the strictness in observing hygienic practices during the manufacturing, as well as to the use of raw materials with good quality and, overall, the effectiveness of the thermal treatment (Aureli et al., 1989; Alamprese et al., 2008; Ricci et al., 2017). No fungi growth and, indeed, no ITS amplicons were retrieved from 2MA pasta samples. As already reported for fresh-filled pasta, molds can be responsible for spoilage, but their multiplication can be inhibited using a concentration of CO_2_ higher than 15% in MAP packaging (Zardetto, 2005b; Tabanelli, 2020). Consistent with these results, AFTs, DON, OTA, and ZEA fungi mycotoxins did not exceed during storage, as they were below the maximum limits established by Commission regulation (No. 401/2006). Mycotoxins are natural food contaminants and the quality and safety of the final pasta depend on the flours, the technological process (thermal treatment and drying process), and the conditions used during preparation (de Nijs et al., 2016; Bouafifssa et al., 2018). The metagenomics explored mycotoxins-producers’ fungi such as *Penicillium*, *Alternaria*, and *Fusarium* being below the 0.1% of relative abundance. The present technological process did not avoid the presence of mycotoxigenic fungal taxa even though mycotoxin production was always under the safety limit confirming the inhibitory effect of MAP toward these important chronic dietary risk factors (Bouafifssa et al., 2018). Living microbes were macroscopically found in 1MA-T90; however, chemical and proteomic analyses did not emphasize a considerable metabolism of macronutrients and proteins for which microbes are well known (De Angelis et al., 2021). The proteomic profiles of all 1MA samples overlapped for both the presence and intensity of gel bands, as well as, albumins, gliadins, and glutenins spots. For this reason, 2MA samples were not profiled with respect to the proteomic field. According to the ITS1-seq, the time of storage determined an increase in alpha diversity in 1MA samples. On the contrary, samples of 1MA were characterized by lower alpha diversity scores of bacteria domains due to the main presence of *Leuconostoc* (at T0), while *Weissella* (at T90) in relative microbiota. Probably due to lower microbial competition, the presence of only these two bacterial genera was not sufficient to avoid mold overgrowth. The presence of ASVs belonging to *Lactobacillus*, *Bacillus*, and *Bifidobacterium* confirmed the addition of BCs (*L. acidophilus*, *L. casei*, *Bifidobacterium* spp., *B. coagulans*) and that these taxa were detectable till 110 days of storage. Therefore, this result confirms the previously stated evidence that probiotic multistrain cultures used for bioprotection are metabolically active preparations applied to inhibit undesired microbes (Spanu et al., 2017). Herein, culturomics highlighted the thermal treatment effectiveness in decreasing cell densities of lactobacilli and bifidobacteria, whereas the spore-forming *B. coagulans* preserved its viability during pasta production and storage as previously discussed (Konuray and Erginkaya, 2020). In the present study, the addition of BCs reduced the growth of autochthonous microbes allowing for improved stability of pasta during storage. This may be attributed to the release of heat-stable bacteriocins synthesized by BCs, which represent an extra hurdle against spoilage and/or pathogen microorganisms during the processing and/or storage and/or selling of the foodstuff (Baka et al., 2014). Antimicrobials could be present in 2MA-BC fresh pasta independently of the viability of the producing microorganisms while contributing to inhibiting the growth of spoilage and pathogenic bacteria and allowing for extending the storage of pasta up to 120 days.

Concerning VOCs, 42 compounds were overall detected in fresh pasta samples. As shown by the multivariate analyses of VOC profiles, samples were mainly differentiated based on the production process (Figure 4). The main differences were related to aldehydes, fatty acids, and alcohols derived from lipid oxidation. Nonanoic and hexanoic acids, both deriving from autoxidation (Giannetti et al., 2014), were significantly abundant in control pasta (1MA-T90) rather than in 2MA-T90 and 2MA-BC-T90 samples. Hence, the experimental MAP substantially extended the shelf-life, probably also delaying lipid oxidation as a result of a reduced O_2_ content (Kimbuathong et al., 2020). Lipid oxidation processes are one of the most important parameters influencing the shelf-life of products affecting their quality through deteriorative events (Calligaris et al., 2007; Difonzo et al., 2018). Microorganisms can synthesize lipase and phospholipase contributing to an increase in free fatty acids, molecules that are susceptible to oxidation, such as aldehydes (Nirmal and Benjakul, 2011). In a similar line, hexanal, which is known as a low-quality marker for artisanal and industrial pasta being responsible for fresh pasta off-flavors, decreased when the CO_2_:N_2_ = 40:60 MAP system and BCs were used (Giannetti et al., 2014; Hong et al., 2021). It is also well known that fungal growth leads to VOC metabolism (Sunesson et al., 1997). With this respect, 2-methylpentanoic anhydride, being a recognized fungal metabolite, was only found in 1MA samples that, in turn, were characterized by the greatest fungal spoilage (Ewen et al., 2004).

## Conclusion

This research highlights the novel exploitation of MAP with or without the addition of BCs to produce fresh pasta. The results indicate that the MAP (40:60 CO_2_:N_2_), high barrier packaging, and BCs and their metabolites acted in a synergistic way to control the microbial spoilage of fresh pasta during refrigerated storage at 4°C and can be introduced at the industrial level giving an increase of 30 days in shelf life compared to low barrier conventional MAP packaging, with potential benefits on the economy and on the environment, spurring innovation in existing production models. The microbial assembly and function varied depending on gas concentration, affecting the microbial stability of fresh pasta. The multi-omics approach used in this study can be applied, combined with traditional protocols, to evaluate which of the studied factors is the most influential to drive the shelf-life of fresh pasta. Even if the added BCs were not all detected by traditional protocols during storage of fresh pasta, their antimicrobials and/or fermentation products leads to quantitative and qualitative effect on the bacterial and fungal microbiota associated with pasta. Data herein reported are encouraging additional tests aimed at setting up new protocols for the use of BCs also in other types of cereal-based products, considering the possible implication that viable microorganisms resting in foods can exert potential positive effects on the gut microbiota of consumers, with consequences that remain to explore.

## Data availability statement

The data presented in this study are deposited in the SRA repository, accession number BioProject PRJNA782807 (https://www.ncbi.nlm.nih.gov/bioproject/PRJNA782807).

## Author contributions

MM, GP, FD, and MDA: conceptualization. MM, MC, GP, FD, and MDA: methodology. MM and MC: validation. MM, MC, GC, GRC, BF, and MV: formal analysis. MM, MC, GRC, GC, BF, DD, MV, and EN: investigation and writing—original draft preparation. FD, GP, and MDA: resources. MM, MC, GRC, GC, BF, DD, and MV: data curation. MM, GRC, GC, BF, and MV: visualization. GP, FD, and MDA: supervision and funding acquisition. GP and FD: project administration. All authors: writing—review and editing.

## Footnotes

1
http://www.bioinformatics.babraham.ac.uk/projects/fastqc/

## Appendix A

After dough making, the 1MA, 2MA, and 2MA-BC fresh pasta samples were conveyed in a steel chamber equipped with a perforated steel conveyor belt. By steam injection at 110 ± 1°C for 90 s, the first step of pasteurization (SARP srl, Padua, Italy, model SST200/V) on the loose product was carried out. After pasteurization, the product was conveyed on a second perforated steel conveyor belt, eliminated from the condensed vapor on the surface, and then cooled to 4°C. Two modified atmospheric conditions and polymeric films with different characteristics as packaging bags were used. In the first, fresh pasta samples obtained by the conventional protocol were packed under MAP and film conventionally used in the semi-industrial factory (1MA). To realize the 1MA headspace conditions, the following gas concentrations were used: 20% CO_2_ and 80% N2 (CO_2_:N_2_ = 20:80), using sealed plastic bags polyamide/polyethylene (PACT30/PE50) (Carton Pack, R&D LAB, Rutigliano, Bari, Italy). The film was characterized by a water vapor permeability (WVTR) of 10 g/m^2^/24 h (38°C, 90% R.H.) and oxygen permeability (OTR) of 20 cc/m^2^/24 h (23°C, 0% R.H.). In the second MAP condition (experimental), fresh pasta samples obtained by the conventional protocol (2MA) or by the addition of the bioprotective mixture (2MA-BC) were packed in PA15/PEEVOH5/PE60 film (Carton Pack) under a modified atmosphere (CO_2_:N_2_ = 40:60). The film was characterized by a WVTR <5 g/m^2^/24 h (38°C, 90% R.H.) and an OTR <2 cc/m^2^/24 h (23°C, 0% R.H.).
