# Supplementary material for: Extension of the shelf-life of fresh pasta using modified atmosphere packaging and bioprotective cultures
Source: Front Microbiol. 2022 Sep 2;13:1003437. doi: 10.3389/fmicb.2022.1003437 (PMC9666361; doi:10.3389/fmicb.2022.1003437)
Supplement: Supplementary file 1 [file Table_1.DOCX]

**Supplementary Table S1.** Concentration (μg/gr) of volatile organic compounds (VOCs) detected and identified in fresh pasta. 1MA, control fresh pasta obtained by conventional protocol and packaging MAP conditions used at plant level; 2MA, fresh pasta obtained by conventional protocol and packaged in experimental MAP conditions; 2MA-BC, fresh pasta obtained by the addition of bioprotective culture onto semolina and packaged in experimental MAP conditions. Samples were analyzed at the beginning (0 days) and the end of the actual 90 days of storage.

| **Compounds** | **Sample Time** | **1MA** | **2MA** | **2MA-BC** |
| --- | --- | --- | --- | --- |
| 2-methyl-pentanoic acid anhydride | T0 | 0.01±0.009a | nd | nd |
|  | T90 | 0.03±0.005a | nd | nd |
| Hexanoic acid | T0 | 0.13±0.001a | 0.07±0.004c | 0.09±0.006b |
|  | T90 | 0.18±0.049a | 0.07±0.001b | 0.09±0.012b |
| Octanoic acid | T0 | nd | 0.01±0.003a | 0.01±0.001a |
|  | T90 | nd | 0.01±0.001a | 0.01±0.001a |
| Nonanoic acid | T0 | 0.01±0.006a | nd | 0.03±0.022a |
|  | T90 | 0.07±0.013a | nd | nd |
| Tot Acids (µg/gr) | T0 | 0.16±0.004 | 0.08±0.007 | 0.12±0.017 |
|  | T90 | 0.28±0.042 | 0.08±0.001 | 0.1±0.013 |
| Ethanol | T0 | nd | 0.02±0.007a | 0.01±0.007b |
|  | T90 | nd | 0.03±0.002a | 0.02±0.006b |
| 1-Pentanol | T0 | 0.05±0.011 | 0.04±0.016 | 0.05±0,001* |
|  | T90 | 0.07±0.011 | 0.03±0.007 | 0.03±0.003* |
| 1-Hexanol | T0 | 0.04±0.001b | 0.08±0.001a | 0.08±0.008a |
|  | T90 | 0.05±0.011 | 0.08±0.003 | 0.07±0.005 |
| 3,5-Octadien-2-ol | T0 | 0.06±0.014 | 0.05±0.002 | 0.04±0.002 |
|  | T90 | 0.09±0.017 | 0.04±0.007 | 0.03±0.002 |
| 3-Decanol | T0 | 0.05±0.012 | 0.06±0.036 | 0.09±0.014* |
|  | T90 | 0.11±0.029 | nd | nd* |
| 1-Octen-3-ol | T0 | 0.32±0.027 | 0.19±0.011 | 0.18±0.036 |
|  | T90 | 0.31±0.026 | 0.25±0.017 | 0.29±0.041 |
| 1-Octanol | T0 | 0.05±0.003 | 0.06±0.034 | 0.05±0.011 |
|  | T90 | 0.04±0.004 | 0.05±0.007 | 0.05±0.005 |
| 2-Octen-1-ol | T0 | 0.07±0.008 | 0.05±0.012 | 0.05±0.003 |
|  | T90 | 0.07±0.007 | 0.06±0.008 | 0.06±0.008 |
| 1-Nonanol | T0 | 0.02±0.002 | 0.04±0.026 | 0.03±0.002 |
|  | T90 | 0.02±0.003 | 0.04±0.005 | 0.03±0.009 |
| Benzyl alcohol | T0 | nd | nd | 0.03±0.003a |
|  | T90 | nd | nd | 0.02±0.003a |
| Phenylethyl Alcohol | T0 | 0.01±0.002 | 0.02±0.008 | 0.02±0.002 |
|  | T90 | 0.01±0.002 | 0.02±0.004 | 0.01±0.003 |
| 2-Hexyl-1-octanol | T0 | 0.01±0.004 | 0.01±0.003 | 0.01±0.003 |
|  | T90 | 0.03±0.019 | nd | nd |
| Phenol, 2,5-bis(1,1-dimethyl ethyl) | T0 | nd | 0.01±0.001 | 0.01±0.001 |
|  | T90 | 0.01±0.001 | 0.01±0.002 | 0.01±0.002 |
| Tot Alcohols(µg/gr) | T0 | 4.38±0.058 | 4.46±0.084 | 4.5±0.063 |
|  | T90 | 5.11±0.179 | 4.68±0.273 | 5.17±0.138 |
| Hexanal | T0 | 0.15±0.026a | 0.18±0.011a* | 0.09±0.003b |
|  | T90 | 0.23±0.031a | 0.11±0.003b* | 0.06±0.015c |
| Heptanal | T0 | 0.02±0.019 | 0.06±0.001 | nd |
|  | T90 | 0.03±0.004 | 0.03±0.011 | 0.02±0.018 |
| 2-Heptenal | T0 | 0.06±0.004 | 0.09±0.035 | 0.05±0.003 |
|  | T90 | 0.09±0.019 | 0.06±0.008 | 0.03±0.008 |
| Nonanal | T0 | 0.29±0.028 | 0.59±0.242 | 0.45±0.069 |
|  | T90 | 0.43±0.095 | 0.27±0.008 | 0.2±0.052 |
| 2-Octenal | T0 | 0.07±0.009 | 0.11±0.021 | 0.08±0.004 |
|  | T90 | 0.11±0.016 | 0.09±0.001 | 0.05±0.014 |
| Decanal | T0 | 0.09±0.038 | 0.21±0.077 | 0.07±0.034 |
|  | T90 | 0.07±0.039 | 0.05±0.018 | 0.08±0.022 |
| 2-Nonenal | T0 | 0.26±0.014b | 0.47±0.062a | 0.38±0.016a |
|  | T90 | 0.31±0.042 | 0.28±0.018 | 0.26±0.044 |
| 2-Octenal, 2-butyl- | T0 | 0.02±0.001 | 0.01±0.007 | 0.01±0.007 |
|  | T90 | 0.02±0.002a | 0.01±0.001b | 0.01±0.006b |
| 2,4-Nonadienal | T0 | nd | 0.02±0.008 | nd |
|  | T90 | nd | nd | nd |
| 2,4-Decadienal | T0 | 0.02±0.002b | 0.13±0.013a* | 0.03±0.004b |
|  | T90 | 0.02±0.004b | 0.05±0.006a* | 0.02±0.005b |
| Tot Aldehydes(µg/gr) | T0 | 0.97±0.059 | 1.84±0.476 | 1.17±0.074 |
|  | T90 | 1.3±0.251 | 0.96±0.057 | 0.73±0.149 |
| Propanedioic acid, diethyl ester | T0 | nd | nd | 0.1±0.012a |
|  | T90 | nd | nd | 0.08±0.007a |
| Octyl ether | T0 | 0.01±0.011 | 0.01±0.008 | 0.02±0.016 |
|  | T90 | 0.03±0.019 | nd | nd |
| Tot Esters (µg/gr) | T0 | 0.01±0.011 | 0.01±0.008 | 0.12±0.028 |
|  | T90 | 0.03±0.019 | nd | 0.08±0.007 |
| Furan, 2-pentyl- | T0 | 0.79±0.065 | 0.8±0.1271 | 0.81±0.038 |
|  | T90 | 0.91±0.115 | 0.98±0.159 | 0.88±0.086 |
| Heptane, 2,2,4,6,6-pentamethyl | T0 | 0.12±0.028a | 0.06±0.001ab | 0.05±0.007b |
|  | T90 | 0.15±0.007a | 0.1±0.021b | 0.06±0.007c |
| 2,2,4,4-Tetramethyloctane | T0 | 0.01±0.006a | nd | nd |
|  | T90 | 0.02±0.003a | 0.01±0.004b | nd |
| Dodecane | T0 | 0.63±0.042 | 0.35±0.336 | 0.64±0.009 |
|  | T90 | 0.63±0.017 | 0.65±0.015 | 0.67±0.004 |
| trans-1-Butenylcyclopentane | T0 | 0.02±0.022c | 0.14±0.002a* | 0.07±0.004b |
|  | T90 | 0.06±0.008a | 0.06±0.012a* | 0.05±0.006a |
| Dodecane, 2,6,10-trimethyl- | T0 | 0.06±0.002a* | 0.01±0.014c | 0.03±0.029bc |
|  | T90 | 0.08±0.003a* | 0.01±0.012b | nd |
| Decane, 3-ethyl-3-methyl- | T0 | 0.04±0.002a | 0.02±0.021a | 0.02±0.021 |
|  | T90 | 0.04±0.001a | nd | nd |
| Decane, 3,8-dimethyl- | T0 | nd | 0.01±0.014a | nd |
|  | T90 | 0.03±0.001a | nd | nd |
| 3,5-Octadien-2-one | T0 | 0.03±0.001a | nd | 0.02±0.001b |
|  | T90 | 0.07±0.011a | 0.01±0.012b | 0.01±0.011b |
| Tot Hydrocarbons(µg/gr) | T0 | 0.89±0.053 | 0.59±0.289 | 0.83±0.037 |
|  | T90 | 1.06±0.049 | 0.86±0.076 | 0.79±0.008 |
| Benzene, m-di-tert-butyl- | T0 | 0.09±0.021 | 0.07±0.071 | 0.03±0.034 |
|  | T90 | 0.09±0.013a | 0.02±0.022b | 0.01±0.012b |
| 5,9-Undecadien-2-one, 6,10-dimethyl ethyl | T0 | 0.03±0.001 | 0.03±0.012 | 0.03±0.011 |
|  | T90 | 0.05±0.01 | 0.02±0.002 | 0.02±0.002 |
| Amylbutyrolactone | T0 | 0.02±0.001b | 0.03±0.003a | 0.03±0.001a |
|  | T90 | 0.02±0.002a | 0.03±0.004a | 0.03±0.005a |
| 2H-Pyran-2,6(3H)-dione | T0 | 0.02±0.001a | nd | nd |
|  | T90 | 0.02±0.003a | nd | nd |
| Tot Others(µg/gr) | T0 | 0.16±0.019 | 0.12±0.086 | 0.09±0.044 |
|  | T90 | 0.18±0.029 | 0.07±0.028 | 0.05±0.005 |

nd, not detected

a-c Values in the same row with different superscript letters differ significantly (p < 0.05)

* Values in the same column differ significantly comparing sample times (p < 0.05)
