# Supplementary material for: Extension of the shelf-life of fresh pasta using modified atmosphere packaging and bioprotective cultures
Source: Front Microbiol. 2022 Sep 2;13:1003437. doi: 10.3389/fmicb.2022.1003437 (PMC9666361; doi:10.3389/fmicb.2022.1003437)

Supplementary Figure 1: Fresh pasta "trofie" during storage at 4 °C. 1MA, control fresh pasta obtained by protocol and packaging MAP conditions used at plant level analyzed at the beginning, after 30, 60 days and at the end of the actual 90 days of shelf life (1MA-T0, 1MA-T30, 1MA-T60, 1MA-T90); 2MA, fresh pasta obtained by conventional protocol and packaged in experimental MAP conditions analyzed at the beginning and the end of the actual and expected 90, 110 and 120 days of shelf life (2MA-T0, 2MA-T90, 2MA-T110, 2MA-T120); 2MA-BC, fresh pasta obtained by the addition of bioprotective culture onto semolina and packaged in experimental MAP conditions, analyzed at the beginning and the end of the actual and expected 90, 110 or 120 days of shelf life (2MA-BC-T0, 2MA-BC-T90, 2MA-BC-T110, 2MA-BC-T120).

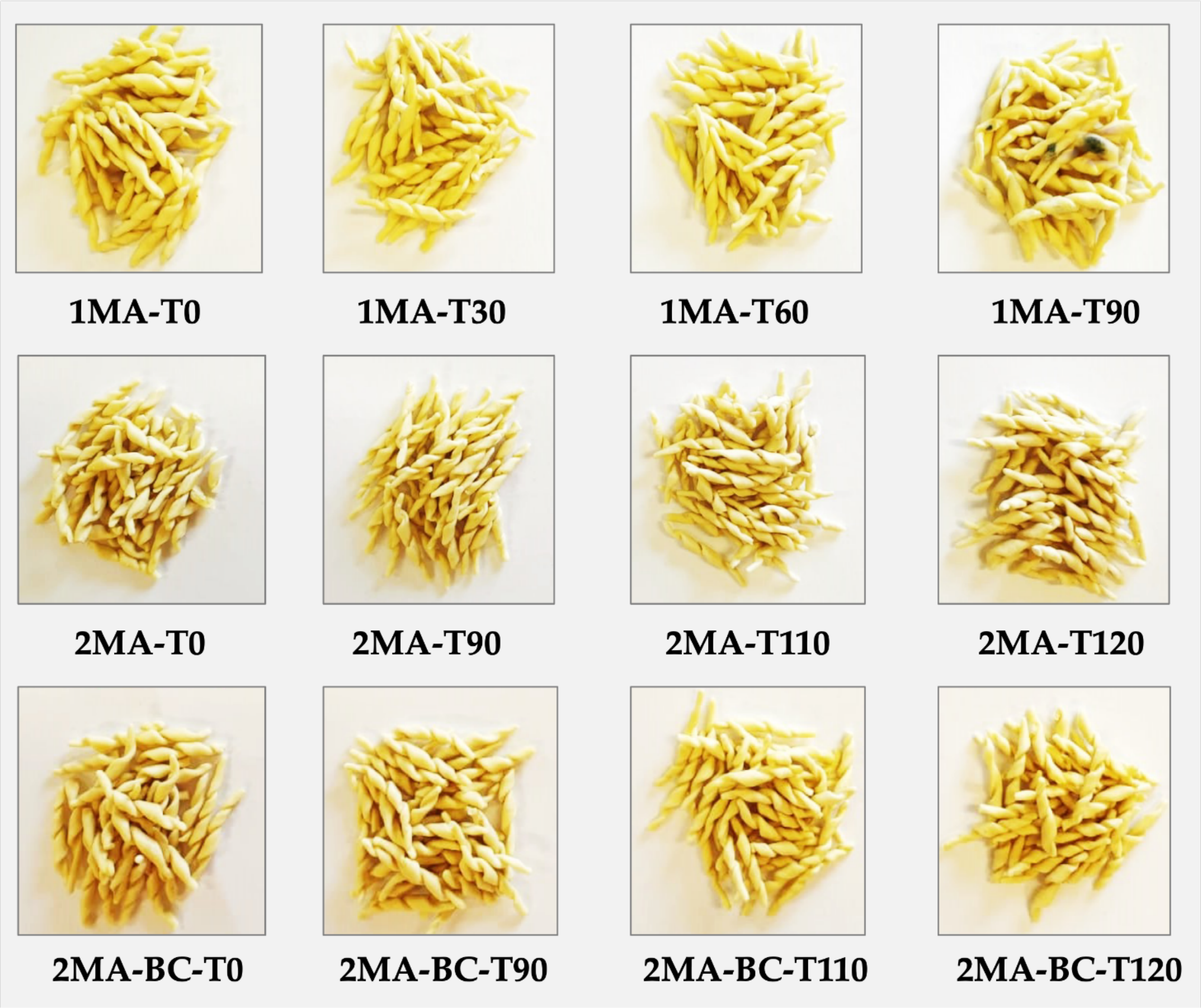

Supplement: Supplementary file 2 [file Image_1.pdf]
