# Supplementary material for: Extension of the shelf-life of fresh pasta using modified atmosphere packaging and bioprotective cultures
Source: Front Microbiol. 2022 Sep 2;13:1003437. doi: 10.3389/fmicb.2022.1003437 (PMC9666361; doi:10.3389/fmicb.2022.1003437)

Supplementary Figure 2: Sodium dodecyl sulfate (SDS)-polyacrylamide gel electrophoresis (PAGE) analysis of the protein fractions (albumins, gliadins and glutenins) extracted from 1MA fresh pasta samples at the beginning, after 30, 60 days and at the end of the actual 90 days of shelf life (1MA-T0, 1MA-T30, 1MA-T60, 1MA-T90). Marker was labeled as M. 1MA composition is described in Materials and Methods.

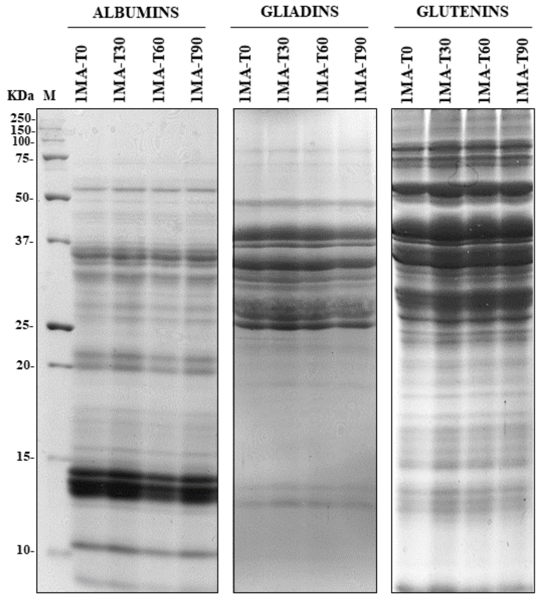

Supplement: Supplementary file 3 [file Image_2.pdf]
